# Patient-led active tuberculosis case-finding in the Democratic Republic of the Congo 

**DOI:** 10.2471/BLT.17.203968

**Published:** 2018-06-04

**Authors:** Emmanuel André, Olivier Rusumba, Carlton A Evans, Philippe Ngongo, Pasteur Sanduku, Marhegane Munguakonkwa Elvis, Habimana Ndwanyi Celestin, Ishara Rusumba Alain, Eric Mulume Musafiri, Jean-Pierre Kabuayi, Olivier le Polain de Waroux, Nadia Aït-Khaled, Michel Delmée, Francis Zech

**Affiliations:** a Department of Microbiology and Immunology, KU Leuven, Herestraat 49, Box 1030, 3000 Leuven, Belgium.; b Ambassadeurs de Lutte Contre la Tuberculose, Bukavu, Democratic Republic of Congo.; c Section of Infectious Diseases and Immunity, Imperial College London, London, England.; d Coordination Provinciale Lèpre et Tuberculose du Sud-Kivu, Bukavu, Democratic Republic of Congo.; e Challenge TB, United States Agency for International Development, United States of America.; f Department of Infectious Disease Epidemiology, London School of Hygiene and Tropical Medicine, London, England.; g International Union against Tuberculosis and Lung Disease, Paris, France.; h Institut de Recherche Expérimentale et Clinique, Université Catholique de Louvain, Brussels, Belgium.

## Abstract

**Objective:**

To investigate the effect of using volunteer screeners in active tuberculosis case-finding in South Kivu, the Democratic Republic of the Congo, especially among groups at high risk of tuberculosis infection.

**Methods:**

To identify and screen high-risk groups in remote communities, we trained volunteer screeners, mainly those who had themselves received treatment for tuberculosis or had a family history of the disease. A non-profit organization was created and screeners received training on the disease and its transmission at 3-day workshops. Screeners recorded the number of people screened, reporting a prolonged cough and who attended a clinic for testing, as well as test results. Data were evaluated every quarter during the 3-year period of the intervention (2014–2016).

**Findings:**

Acceptability of the intervention was high. Volunteers screened 650 434 individuals in their communities, 73 418 of whom reported a prolonged cough; 50 368 subsequently attended a clinic for tuberculosis testing. Tuberculosis was diagnosed in 1 in 151 people screened, costing 0.29 United States dollars (US$) per person screened and US$ 44 per person diagnosed. Although members of high-risk groups with poorer access to health care represented only 5.1% (33 002/650 434) of those screened, they contributed 19.7% (845/4300) of tuberculosis diagnoses (1 diagnosis per 39 screened). The intervention resulted in an additional 4300 sputum-smear-positive pulmonary tuberculosis diagnoses, 42% (4 300/10 247) of the provincial total for that period.

**Conclusion:**

Patient-led active tuberculosis case-finding represents a valuable complement to traditional case-finding, and should be used to assist health systems in the elimination of tuberculosis.

## Résumé

**Objectif:**

Étudier les effets de l'utilisation d'examinateurs volontaires dans le dépistage de la tuberculose active au Sud-Kivu, en République démocratique du Congo, en particulier dans les groupes exposés à un risque élevé d'infection tuberculeuse.

**Méthodes:**

Afin d'identifier et d'examiner des groupes à haut risque dans des communautés isolées, nous avons formé des examinateurs volontaires, qui étaient principalement des individus ayant eux-mêmes reçu un traitement contre la tuberculose ou ayant un antécédent familial de la maladie. Une organisation à but non lucratif a été créée et les examinateurs ont reçu une formation sur la maladie et sa transmission dans le cadre d'ateliers de 3 jours. Les examinateurs ont enregistré le nombre de personnes examinées, faisant état d'une toux prolongée et qui se sont rendues dans un centre de consultation pour bénéficier d'un dépistage, ainsi que les résultats des examens. Les données ont été évaluées tous les trimestres pendant les 3 ans qu'a duré l'intervention (2014-2016).

**Résultats:**

L'acceptabilité de l'intervention était élevée. Les volontaires ont examiné 650 434 membres de leurs communautés, parmi lesquels 73 418 ont fait état d'une toux prolongée; 50 368 se sont ensuite rendus dans un centre de consultation pour bénéficier d'un dépistage de la tuberculose. La tuberculose a été diagnostiquée chez 1 personne examinée sur 151, pour un coût de 0,29 dollar des États-Unis (US$) par personne examinée et 44 US$ par personne diagnostiquée. Bien que les membres des groupes à haut risque qui ont un accès plus restreint aux soins de santé ne représentaient que 5,1% (33 002/650 434) des personnes examinées, ils représentaient 19,7% (845/4300) des cas de tuberculose diagnostiqués (1 personne diagnostiquée sur 39 personnes examinées). L'intervention a donné lieu à 4300 autres diagnostics de la tuberculose pulmonaire à frottis positif, soit 42% (4300/10 247) du total de la province pour la période considérée.

**Conclusion:**

Le dépistage de la tuberculose active par les patients complète utilement le dépistage traditionnel et devrait être utilisé pour aider les systèmes de santé à éradiquer la tuberculose.****

## Resumen

**Objetivo:**

Investigar el efecto del uso de examinadores voluntarios para la detección de casos de tuberculosis activa en Kivu del Sur (República Democrática del Congo), sobre todo entre los grupos de alto riesgo de infección por tuberculosis.

**Métodos:**

Para identificar y examinar los grupos de alto riesgo en comunidades remotas, hemos formado a examinadores voluntarios, principalmente aquellos que habían recibido tratamiento para la tuberculosis o tenían antecedentes familiares de la enfermedad. Se creó una organización sin ánimo de lucro y los examinadores recibieron formación sobre la enfermedad y su transmisión en talleres de tres días. Los examinadores registraron el número de personas examinadas que mostraban una tos prolongada y que acudían a una clínica para hacerse la prueba, así como los resultados de la prueba. Los datos se evaluaron cada trimestre durante el periodo de tres años de la intervención (2014-2016).

**Resultados:**

La aceptación de la intervención fue elevada. Los voluntarios examinaron a 650 434 personas en sus comunidades, 73 418 de las cuales presentaron una tos prolongada; posteriormente, 50 368 acudieron a una clínica para someterse a pruebas de tuberculosis. Se diagnosticó tuberculosis a 1 de cada 151 personas examinadas, lo que costó 0,29 USD por persona examinada y 44 USD por persona diagnosticada. Aunque los miembros de los grupos de alto riesgo con peor acceso a la atención de la salud representaban solo el 5,1 % (33 002/650 434) de los examinados, supusieron el 19,7% (845/4300) de los diagnósticos de tuberculosis (1 diagnóstico por cada 39 examinados). La intervención dio como resultado 4300 diagnósticos adicionales de tuberculosis pulmonar con frotis de esputo positivo, el 42% (4300/10 247) del total provincial durante el periodo.

**Conclusión:**

La búsqueda de casos de tuberculosis activa dirigida por el paciente representa un complemento valioso a la búsqueda de casos tradicional y debería utilizarse para ayudar a los sistemas sanitarios a eliminar la tuberculosis.

## ملخص

**الهدف:**

دراسة تأثير الاستعانة بأفراد فحص متطوعين في اكتشاف حالات السل النشطة في جنوب كيفو، بجمهورية الكونغو الديمقراطية، وخاصة بين المجموعات المعرضة لخطر مرتفع للإصابة بالسل.

**الطريقة:**

بهدف ****التعرف على المجموعات المعرضة لخطر مرتفع في المجتمعات النائية وفحصها، قمنا بتدريب بأفراد فحص متطوعين، وخاصة أولئك الذين تلقوا العلاج من مرض السل أو لديهم تاريخ عائلي للمرض. تم إنشاء منظمة غير هادفة للربح، وحصل أفراد الفحص على تدريب على المرض وكيفية انتقال العدوى الخاصة به، في حلقات عمل استمرت لمدة ثلاثة أيام. سجل أفراد الفحص عدد الأشخاص الذين تم فحصهم، والذين أفادوا بوجود سعال لفترة ممتدة، وحضروا للعيادة للخضوع للكشف، فضلاً عن نتائج الكشف. تم تقييم البيانات كل ثلاثة أشهر خلال فترة التدخل التي استمرت لمدة 3 سنوات (2014 إلى 2015).

**النتائج:**

كانت مقبولية التدخل عالية. قام المتطوعون بفحص 650434 فرداً في مجتمعاتهم المحلية، وأفاد 73418 منهم بوجود سعال طويل؛ أتى 50368 منهم لاحقا للعيادة لإجراء اختبار للكشف عن مرض السل. تم تشخيص الإصابة بمرض السل في 1 من بين 151 شخصا تم فحصهم، بتكلفة قدرها 0.29 دولار أمريكي لكل شخص تم فحصه، و 44 دولاراً أمريكياً للشخص الذي تم تشخيص إصابته بالمرض. على الرغم من أن أعضاء المجموعات المعرضة لخطر كبير، والذين يعانون من مستوى أقل من الحصول على الرعاية الصحية، لم يمثلوا سوى 5.1٪ (33002/650434) من الأشخاص الذين تم فحصهم، إلا أنهم قد ساهموا بنسبة 19.7٪ (845/4300) من تشخيصات السل (تشخيص واحد لكل 39 فحصاً). أدى التدخل إلى عدد 4300 تشخيصاً إضافياً بمرض السل الرئوي بفحص مسحة اختبار البصاق، بنسبة 42٪ (4300/10247) من إجمالي المقاطعات لتلك الفترة.

**الاستنتاج:**

يمثل اكتشاف حالات السل النشط بين المرضى عنصر تكميلي قيّم للكشف التقليدي عن الحالات، وينبغي الاستعانة به لمساعدة النظم الصحية في القضاء على مرض السل. ****

## 摘要

**目的:**

研究使用疾病筛查志愿者对于在刚果民主共和国南基伍省，尤其是结核病感染高风险群体中，发现开放性结核病的病例的作用。

**方法:**

为识别和筛选偏远地区的高风险群体，我们对疾病筛查志愿者进行培训，主要是那些自己接受过结核病治疗或有此疾病家族史的人。我们创建了一个非营利组织，疾病筛查人员在为期 3 天的研讨会中接受了针对此疾病及其传播的培训。疾病筛查人员记录被筛查人员数，报告持续咳嗽人员、参与临床测试人员和测试结果。在为期 3 年（2014-2016 年）的干预中，每季度评估一次数据。

**结果:**

干预的接受度非常高。志愿者对其社区中 650434 人进行筛查，其中 73418 人报告有长期咳嗽，50368 人相继参与结核病临床测试。在筛查人群中每 151 人中就有 1 人诊断为结核病，筛查每人花费 0.29 美元 (US$)，诊断每人花费 44 美元。尽管享受医疗服务机会较少的高风险群体仅占所有被筛查人员的 5.1% (33002/650434)，他们却占确诊结核病患者（每 39 位筛查者中就有 1 人诊断）的 19.7% (845/4300)。此干预确诊了另外 4300 例痰涂片阳性肺结核，占该期间省总数的 42% (4300/10247)。

**结论:**

患者主导开放性结核病的病例发现是对传统病例发现的宝贵补充，应用来协助医疗卫生体系消除结核病。****

## Резюме

**Цель:**

Изучить влияние использования добровольцев в качестве специалистов по скрининговому обследованию на степень выявления случаев заболевания активным туберкулезом в провинции Южное Киву, Демократической Республики Конго, особенно среди групп с высоким риском инфицирования туберкулезом.

**Методы:**

Чтобы выявить и отобрать группы с высоким риском инфицирования в отдаленных сообществах, авторы обучили специалистов-добровольцев проведению скринингового обследования (в основном тех, кто сам ранее получал лечение от туберкулеза или имел семейную историю заболевания). Была создана некоммерческая организация, которая организовала трехдневные семинары, где специалисты по скрининговому обследованию прошли курс, посвященный туберкулезу и способам передачи этой инфекции. Специалисты по скрининговому обследованию регистрировали количество людей, прошедших скрининг, лиц с продолжительным кашлем и тех, кто обратился в клинику для тестирования, а также результаты тестирования. Данные оценивались каждый квартал в течение трехлетнего периода вмешательства (2014–2016 гг.).

**Результаты:**

Приемлемость вмешательства была высокой. Волонтеры отобрали 650 434 человека в своих общинах, у 73 418 из которых был отмечен продолжительный кашель; 50 368 впоследствии обратились в клинику для тестирования на туберкулез. Туберкулез был диагностирован у 1 из 151 человека, прошедшего скрининговое обследование. Расходы на проведение скрининга составили 0,29 долл. США на человека, прошедшего скрининговое обследование, и 44 долл. США на человека, у которого туберкулез был подтвержден диагнозом. Несмотря на то что доля людей, входящих в группы повышенного риска с плохим доступом к медицинскому обслуживанию, составила лишь 5,1% (33 002/650 434) из тех, кто прошел скрининговое обследование, эта же доля людей составила 19,7% (845/4300) случаев подтвержденного диагнозом туберкулеза (1 диагноз на 39 прошедших скрининговое обследование). Это вмешательство привело к дополнительным 4300 диагнозам туберкулеза легких с положительным результатом мазка мокроты, что составило 42% (4300/10 247) от общего количества провинций за этот период.

**Вывод:**

Выявление случаев заболевания активным туберкулезом при активном участии самих пациентов представляет собой ценное дополнение к традиционному методу выявления случаев этого заболевания и должно использоваться для оказания помощи системам здравоохранения в борьбе с туберкулезом.

## Introduction

Of the estimated 10.4 million people worldwide who developed tuberculosis in 2016, one third of these were undetected or unreported.^1^ In many so-called high-burden countries,^2^ such as in the Democratic Republic of the Congo, the number of undetected cases exceeds 50%. With a tuberculosis incidence of 323 cases per year per 100 000 inhabitants^1^ and an estimated population of 77 million inhabitants, over 125 000 new cases of tuberculosis in the country go undetected every year.

Previous evaluations of active case-finding interventions have failed to demonstrate their utility and sustainability in programmatic conditions, especially in reaching high-risk groups.^3^^,^^4^ However, a review by the World Health Organization (WHO)^5^ and a later study^6^ revealed that, where baseline tuberculosis risks are high, the impact and cost–effectiveness of active case-finding on tuberculosis detection can also be high. Active case-finding is recommended by WHO for high-risk groups, defined as having: (i) a higher tuberculosis burden than the general population; (ii) limited access to health care; or (iii) a tuberculosis incidence of over 1000 per 100 000 inhabitants per year.^5^

Active case-finding usually involves screening by trained health professionals. Such screeners can however be geographically and socially distant from populations at high risk for the disease screened. To address this issue, peer-led interventions have been piloted and have shown a positive impact on the control of diseases such as human immunodeficiency virus (HIV),^7^^,^^8^ malaria^9^ and tuberculosis.^10^^,^^11^

This study presents an intervention where we trained people who had been directly affected by tuberculosis, either those who had received treatment for tuberculosis or with a family history of tuberculosis, to conduct active case-finding. We expected that many of these people would already belong to the social groups and geographical areas of high risk of tuberculosis, which would facilitate access to these groups. Furthermore, we anticipated that having volunteer screeners who had themselves received tuberculosis treatment would increase empathy, decrease stigmatization and increase the acceptability of screening and tuberculosis testing.

The aims of the study were to investigate the effect of using such volunteer screeners, who would ask people considered to be at high risk of tuberculosis infection about the presence of a prolonged cough (lasting more than 15 days), and encourage attendance at a clinic for testing if they reported this symptom.

## Methods

### Setting

This study took place in the South Kivu province at the eastern border of the Democratic Republic of the Congo. Over two decades of conflicts have profoundly affected the inhabitants of the country,^12^^,^^13^ resulting in a sizeable military population. Further, the exploitation of gold-mining resources by the so-called artisanal mining sector means that thousands live in remote camps with adverse sanitary conditions and little access to health care.^14^ As well as people who live in the same house as others being treated for tuberculosis, the main tuberculosis high-risk environments include prisons,^15^^,^^16^ mining communities^17^ and military camps.^18^^,^^19^

The estimated population of the province in 2015 was 5.8 million inhabitants,^20^ spread over 34 zones containing over 500 health facilities; only 113 of these health facilities provide tuberculosis services (an increase from just 78 until 2014). Ziehl–Neelsen microscopy and first-line anti-tuberculosis drugs are provided free, but patients have to pay for initial medical visits. Ten clinics perform Xpert® MTB/RIF testing, a nucleic acid amplification test to detect *Mycobacterium tuberculosis* nucleic acid and a genetic sequence indicative of rifampicin resistance. This test is primarily used for retreatment patients and those infected with HIV. Tuberculosis diagnosis rarely involves chest radiographs as they are only available in a few clinics and cost patients 20 United States dollars (US$).

### Intervention

The intervention programme began at the end of 2013 and we evaluated results every quarter until the end of 2016.

In 2013, cured patients living in the clinic catchment area were personally contacted and invited to join an information session calling for volunteer screeners. Patients accepting the invitation to become volunteers joined a local screening group, part of a provincial non-profit organization called *Ambassadeurs de Lutte Contre la Tuberculose*. Funded by a TB Reach grant, the organization was created by Olivier Rusumba with the support of Emmanuel André with the aim of organizing the work of the screeners. Box 1 describes the organization in detail. Staff members from the organization and members of the national tuberculosis programme jointly provided a 3-day workshop for the volunteer screeners on the risk factors, symptoms and treatment of tuberculosis, as well as the importance of early detection. At the workshop, we held group discussions on strategies that could be implemented locally to target the people in the community thought to be at greatest risk due to particular living conditions or marginalization; we emphasized how people sharing homes with other tuberculosis patients were especially at risk. Through door-to-door screening, volunteers identified such members of the community and referred them for testing if they reported a prolonged cough, by informing them of the location of local clinics performing free testing. Screeners compiled reports recording four key variables: (i) the number of people screened for the presence of a prolonged cough in their community; (ii) the number and proportion of people identified with a prolonged cough; (iii) the number and proportion of people with a prolonged cough who attended a clinic for testing; and (iv) individual test results regularly collected by the screeners at the local clinic. TB Reach and Challenge TB provided funding to cover administration costs and small incentive payments to volunteers.

Box 1 **Non-profit organization created for tuberculosis active case-finding, Democratic Republic of the Congo, 2014–2016**

People with tuberculosis and their communities in South Kivu created a non-profit organization called *Ambassadeurs de Lutte Contre la Tuberculose*.

**Objectives:**

To support members of tuberculosis-affected households by providing them with socially useful work actively seeking community members with a prolonged cough and referring them for tuberculosis testing. This aimed to reduce morbidity, mortality and contagion.

**Organization:**

Local volunteer groups of 1–20 screeners worked in the catchment area of their local clinic, to which they referred people with a prolonged cough. To create each group, we invited up to 10 cured patients living in the clinic catchment area to participate in a 3-day workshop to learn from clinic staff about: tuberculosis disease; its transmission, treatment and under-detection; groups at high risk; and the need to actively identify and diagnose patients.

**Screening:**

Each volunteer group defined and organized their active case-finding activities, including how to target screening of people at high risk of tuberculosis. Screeners recorded whether those screened belonged to any of the high-risk groups: people residing in a household with a patient receiving tuberculosis treatment or in a prison, mining community or military base.

**Financial incentives:**

Each group received US$ 30 per quarter for administration and transport, and volunteer screeners received US$ 0.50 per symptomatic person who attended for tuberculosis testing. Payments were made after data validation in the quarterly notification report by clinic and provincial staff, subject to the availability of funds. The intervention also paid each clinic US$ 0.50 per diagnostic test performed, to remunerate the workload associated with increased patient referrals.

**Funding:**

The intervention was fully externally funded from the end of 2013 until the end of 2014; reduced external funding continued until the end of 2016.

US$: United States dollars.

### Evaluation

Study objectives included quantifying the number of people within different groups of high risk who needed to be screened to find one patient with sputum-smear-positive pulmonary tuberculosis, as well as determining the acceptability, effectiveness and impact of the active case-finding intervention and assessing its long-term sustainability (Box 2).

Box 2 **Objectives of tuberculosis active case-finding study, Democratic Republic of the Congo, 2014–2016**

1. Acceptability of: the intervention to the community and health systems; participation in active case-finding to people who had received tuberculosis treatment; and attendance at a clinic to undergo testing to those screened who report a prolonged cough.

2. Effectiveness, assessed as: quantification of the number of people who needed to be screened to detect a single case of tuberculosis, including within marginalized populations at high risk of tuberculosis.

3. Impact, observed in: increasing tuberculosis case-finding in the clinics where the intervention took place; actively finding an important proportion of patients before ongoing passive case-finding; and tangibly increasing provincial tuberculosis notification figures.

4. Sustainability, indicated by the ability of clinics to maintain the active-case finding intervention over time.

We collected reports from screeners detailing their activities, and cross-checked these against each clinic’s official notification of the number and identity of patients diagnosed with sputum-smear-positive pulmonary tuberculosis. We calculated the total cost of the intervention, that is, direct activity expenses, including incentives paid to screeners and clinics, and operational and structural costs of *Ambassadeurs de Lutte Contre la Tuberculose*. We divided this cost by the number of people in the different groups, for example screeners, people screened, and people diagnosed with tuberculosis (Table 1). To assess sustainability we defined groups with continuous activity as those reporting at least nine quarters of activity per year, without inactivity lasting longer than one quarter, and for which any single quarter of inactivity was separated by at least two quarters of activity.

**Table 1:** Running costs of the active tuberculosis case-finding intervention, South Kivu, Democratic Republic of the Congo, 2014–2016

Part of intervention	Proportion of cost, %	Total cost, US$		Average spent over the study period, US$					
				Screener (*n* = 775)	Clinic (*n* = 69)	Person screened (*n* = 650 434)	Person with cough (*n* = 73 418)	Person tested (*n* = 50 368)	Per positive diagnosis^a^ (*n* = 4300)
Active screeners	24	44 594		58	646	0.07	0.61	0.89	10
Local health facilities	13	25 184		32	365	0.04	0.34	0.50	6
Field supervision	38	72 000		93	1043	0.11	0.98	1.43	17
Administration	25	46 800		60	678	0.07	0.64	0.93	11
**TOTAL**	**100**	**188 578**		**243**	**2733**	**0.29**	**2.57**	**3.74**	**44**

US$: United States dollars.

^a^ Defined as sputum-smear-positive pulmonary tuberculosis.

Note: These data do not take into account the costs associated with the initial training of the volunteer screeners and the implementation of screening groups, and inconsistencies arise in some totals due to rounding.

Since two sputum-smear microscopy tests were the main tuberculosis testing in this setting, we only considered sputum-smear-positive pulmonary tuberculosis as bacteriologically confirmed tuberculosis.

To better capture the high diversity of results reported between screening groups and the progress of each screening group over time, we analysed quarterly screening and tuberculosis notifications from health facilities as 647 units, with an internal correlation for the successive results per clinic. These results had a negative-binomial gamma–Poisson distribution due to hyperdispersion between health facilities. This procedure, a requirement due to the structure of the data, gives a weight to each unit different from the crude count of the population, minimizing the influence of units with exceptionally high counts. Similarly, the relations between the reported screening activities, tuberculosis diagnoses and time were examined using a generalized linear regression of a negative-binomial gamma–Poisson variable. For percentages, we used a generalized linear regression of a β-binomial variable. We used quasi-least squares, which is suitable for calculating internal correlations to adjust the regression, and assumed a constant between-year correlation matrix. Regression validity was checked by applying an Anscombe transform to residuals. We used the sandwich variance matrix to calculate statistical significance.

This study was an operational audit of programmatic and surveillance anonymized data which were collected as part of the project, and did not require patient consent.

## Results

**Fig. 1:**
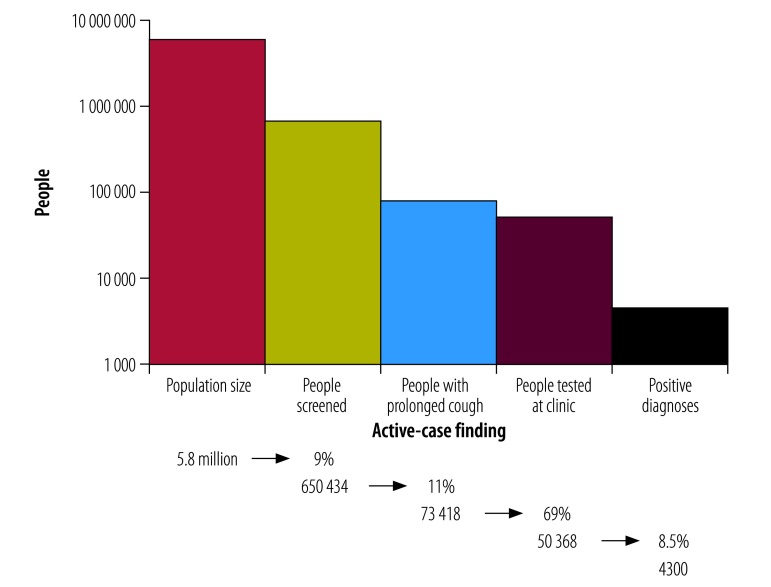
Acceptability of active tuberculosis case-finding, Democratic Republic of the Congo, 2014–2016

**Fig. 2:**
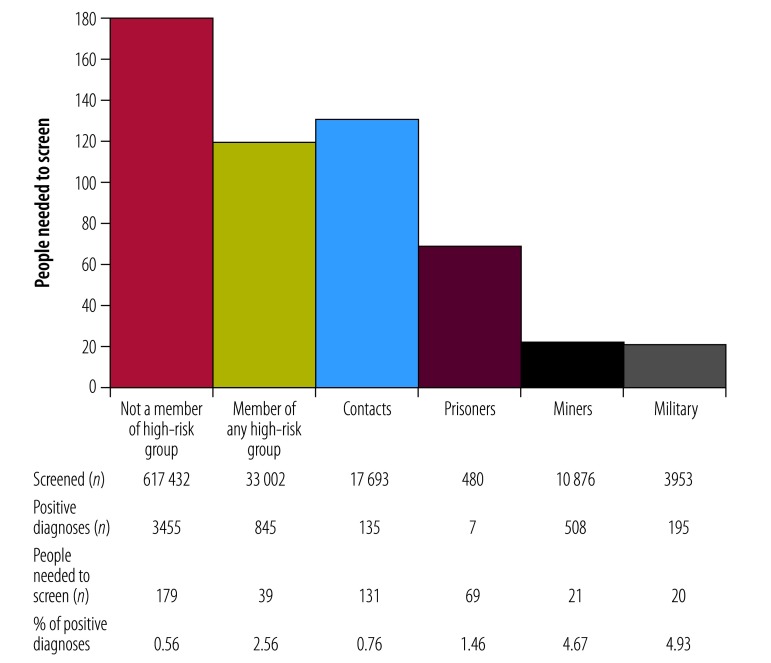
Effectiveness of active tuberculosis case-finding, Democratic Republic of the Congo, 2014–2016

Notes: The number of people needed to screen represents the number who needed to be screened to diagnose a single case of sputum-smear-positive pulmonary tuberculosis. The high-risk group contacts are defined as those living in a household with someone already diagnosed with tuberculosis.

**Fig. 3:**
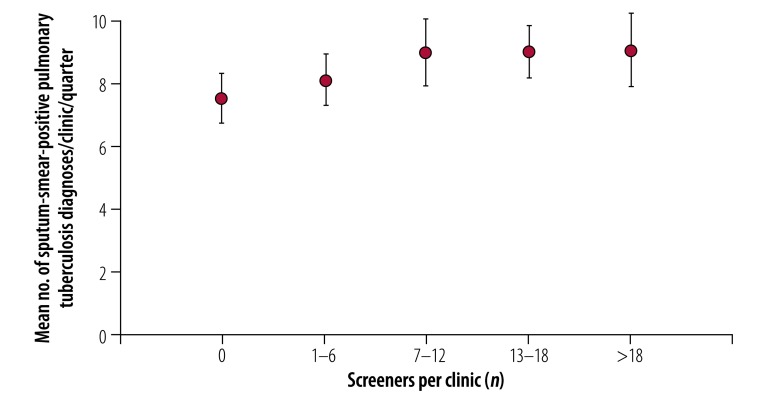
Impact of active tuberculosis case-finding from 113 clinics, Democratic Republic of the Congo, 2014–2016

Note: The plot shows the mean and standard error of the mean.

In total, 1713 volunteers screened 650 434 people, which resulted in 4300 people diagnosed with sputum-smear-positive pulmonary tuberculosis (Fig. 1). Fig. 2 and Fig. 3 show the crude numbers and proportions for effectiveness and impact of the active case-finding intervention, respectively. In the following sections we adjusted the proportions statistically to reduce the effect of very high counts.

### Acceptability

All clinics and communities in the study area accepted the intervention (Fig. 1). On average, 69 groups (range, 24–100) of 775 screeners (range, 197–1713) participated in the intervention each quarter. Over the entire 3-year intervention, an average of 11% (73 418) of individuals screened reported a prolonged cough; 69% (50 368) of those with a prolonged cough attended the clinic and were tested for tuberculosis. We also calculated the average based on the results reported for each clinic and over each quarter. These percentages varied slightly compared with the general figures due to the varying levels of intensity of screening between different areas and over time. Based on this second analytical approach, when each clinic operating over each quarter was considered a separate entity, 14% (95% confidence interval, CI: 13.34–14.66) of individuals screened reported a prolonged cough and 66% (95% CI: 64.33–67.67) of those with a prolonged cough attended the clinic for tuberculosis testing.

### Effectiveness

On average, when each clinic over each quarter was considered as a separate entity, 10% (95% CI: 9.24–10.76) of patients who had tuberculosis testing were diagnosed with sputum-smear-positive pulmonary tuberculosis. This proportion was similar (8.5%) when considering the entire intervention over the 3-year duration. Over the study period, the average yield of a screener was 1.8 new sputum-smear-positive pulmonary tuberculosis cases diagnosed per year. Members of marginalized groups contributed 19.7% (845/4300) of tuberculosis diagnoses (Fig. 2), although they only represented 5.1% (33 002/650 434) of people screened. When each clinic over each quarter was considered as a separate entity, the number who needed to be screened to detect a single case of tuberculosis was 128 (95% CI: 113.9–142.1); this number was 151 when considering the entire intervention as a whole. The number who needed to be screened was significantly (*P* < 0.0001) lower in mining camps (21), military camps (20) and prisons (69). By summing the costs associated with the implementation and training of the screening groups, the cost of the intervention per sputum-smear-positive pulmonary tuberculosis diagnosis was found to be US$ 44 (Table 1).

### Impact

The presence of any screener(s) was associated with more sputum-smear-positive pulmonary tuberculosis diagnoses at the clinic level (*P* = 0.005; Fig. 3) and a greater number of screeners per group resulted in a greater number of diagnoses.

Throughout 2014–2016, 42% (4 300/10 247) of patients diagnosed in the South Kivu province were found through this active case-finding intervention; annually, the intervention was associated with an increase in tuberculosis cases notified at the provincial level (*P* = 0.04). This increase is not statistically significant if adjusted for an estimated 3.1% annual increase in the provincial population, although reliable data were unavailable for changes in population size.

### Sustainability

**Fig. 4:**
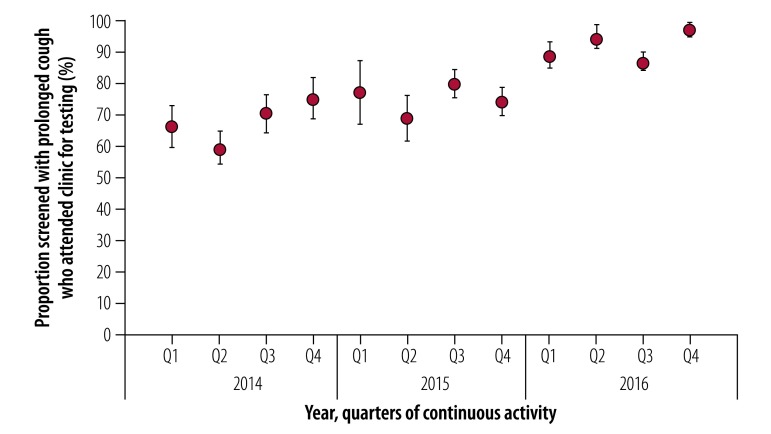
Acceptability of active tuberculosis case-finding within the 24 continuous-activity screening groups, Democratic Republic of the Congo, 2014–2016

Note: The plot shows the mean and standard error of the mean proportion of people screened with a prolonged cough who attended a clinic for testing.

**Fig. 5:**
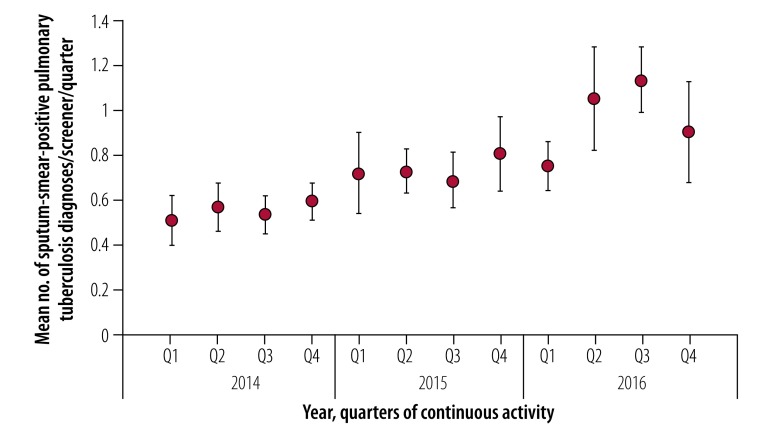
Sustainability of active tuberculosis case-finding within the 24 continuous-activity screening groups, Democratic Republic of the Congo, 2014–2016

Note: The plot shows the mean and standard error of the mean number of sputum-smear-positive pulmonary tuberculosis diagnoses per screener per quarter.

Sustainability was assessed over the 3 years of the intervention for the 24 groups who maintained continuous activity (Fig. 4 and Fig. 5). These groups referred a higher proportion of people screened who were reporting a prolonged cough to clinics for tuberculosis testing compared with the 76 groups who reported interrupted activity during the study (odds ratio: 1.3; 95% CI: 1.3–1.4). While the 24 continuous-activity groups represented 37% (634/1713) of screeners, they reported 36% (234 156/650 434) of people screened, 40% (29 367/73 418) of people reporting a prolonged cough, 47% (23 672/50 368) of those attending a clinic for tuberculosis testing and 59% (2537/4300) of sputum-smear-positive pulmonary tuberculosis diagnoses during the intervention. Acceptability was higher among these continuous-activity groups compared with the average: the mean proportion of people reporting a prolonged cough who attended a clinic for tuberculosis testing (Fig. 4) was 77% (95% CI: 76.6–77.4) with an increase of 50% (95% CI: 33–67) observed between the first and the last quarter. When analysing the individual performance of screeners over the study period (Fig. 5), the number of diagnoses per screener per quarter increased by 118% (95% CI: 70–166) between the first and the last quarter.

## Discussion

This study highlights the utility of performing active case-finding in the populations underserved by the health system of the South Kivu province of the Democratic Republic of the Congo. Using volunteer screeners who had a personal or family history of tuberculosis as active-case finders, organized as a local non-profit organization, probably explains the ability of the screeners to connect with members of high-risk groups and access remote populations. We also demonstrate the increasing effectiveness of these volunteer screeners over time. The low numbers of people who had to be screened to detect a single case of tuberculosis (only 20 in military camps), and the low costs per diagnosis (US$ 44), are compatible with WHO criteria^5^ for recommending active case-finding as a cost–effective, complementary approach to traditional case-finding. The observed number who needed to be screened to detect a single case of tuberculosis may have been even lower if more sensitive diagnostics, such as nucleic acid amplification test or culture had been available, if the clinical staff had been more consistent at diagnosing tuberculosis,^21^ or if chest radiographs had been more widely available.

Levels of acceptability of the intervention by communities, clinics, screeners and those screened were all high. According to local screeners, potential reasons for not accepting a referral for tuberculosis testing may have included: (i) the considerable distance to clinics in rural areas; (ii) limited trust in the volunteer screeners; and (iii) the stigmatizing and impoverishing effects of tuberculosis.

The effectiveness of the present intervention was comparable with active case-finding yield generally reported in populations with an annual incidence of tuberculosis cases greater than 300 per 100 000 people.^5^ Our data show that the active case-finding intervention mainly occurred in households and communities where tuberculosis was actively circulating (contacts in Fig. 2); the number of such individuals who needed to be screened to detect a single case of tuberculosis was 131. This number was even lower among people living in mining and military camps and prisons. This supports WHO policy^5^ that such groups should be prioritized for active case-finding.

The intervention potentially reduced tuberculosis-related morbidity and mortality and reduced transmission of the disease in the community. The number of sputum-smear-positive pulmonary tuberculosis diagnoses increased with the number of screeners involved in each local screening group. The intervention was also associated with increased provincial diagnoses, although this finding is uncertain, because of limited demographic data; the pertinence of evaluating the intervention by provincial notification is, however, limited due to the fact that only a fraction of clinics participated. This non-randomized evaluation was highly informative, however, and should be complemented by future randomized studies.

The 24 screening groups that maintained continuous activity and received undisrupted funding throughout the evaluation demonstrated a higher performance in terms of numbers of screeners, population screened, people identified with a prolonged cough, those tested for tuberculosis and positive diagnoses. Furthermore, in the continuous-activity groups, the number of people screened and tested and the number of new diagnoses all increased over time. These figures suggest that the ability of the volunteer screeners to perform active case-finding improves with experience, supporting longer-term projects. Through the very high number of contacts between the screeners and the population screened (representing approximately 10% of the population), the intervention probably increased awareness of tuberculosis and its symptoms in the general South Kivu population. However, sustainability is also influenced by the ability of the non-profit organizations to secure funding; although we have demonstrated the effectiveness of this community-driven intervention, staff members are not skilled in responding to highly competitive international grant calls and would benefit from international support to publicize their work.

Our study has three main limitations intrinsically associated with the design of the intervention and the non-profit characteristic of *Ambassadeurs de Lutte Contre la Tuberculose*: the high variability among the screening groups; the simplicity of the screening algorithm; and a lack of data on the results of treatment following diagnosis. The high variability among the screening groups regarding the number of people screened and diagnosed with sputum-smear-positive pulmonary tuberculosis could be explained by a variation in the baseline incidence of tuberculosis among different zones and the variability in the clinical skills of local medical staff, as has been suggested elsewhere.^21^ The screening protocol, the presence of a prolonged cough, was selected based on the fact that volunteers would have had little or no medical training. However, this symptom is only present in approximately 35% of patients with pulmonary tuberculosis disease. When followed by a positive smear microscopy or nucleic acid amplification test, only 21–32% of all people screened who actually have tuberculosis are diagnosed.^4^ Further, due to the nature of the clinical test, incidences of smear-negative pulmonary tuberculosis or extra-pulmonary tuberculosis would have remained undiagnosed. Finally, we did not design this study to assess the outcome of patients undergoing treatment or to evaluate any differences in patient outcomes between those referred through active case-finding compared with passive referral.

The challenges faced during this intervention were the reporting and supervision systems required to monitor the activities of and provide support for screening groups facing problems. As detailed in Table 1, supervision represented 38% of the US$ 44 cost of each sputum-smear-positive pulmonary tuberculosis diagnosis; reaching screening groups who were scattered over the large province incurred travel costs for supervisors. The paper-based recording and dissemination of screening reports also represented a large proportion of the total cost (25%) of each diagnosis. Providing these groups with connected and digitalized reporting systems may decrease such costs in the future.

Our results suggest that if peer-led interventions can be funded long-term, that is, sustained, they can represent a valuable complement to the traditional and passive case-finding in the global effort to control tuberculosis. We propose that patients be encouraged to engage in active case-finding interventions at their local level; the observed improvement over time in the ability of such volunteer screeners among the most vulnerable, could help to reduce the costs associated with tuberculosis,^22^ while potentially contributing to its elimination.
